# Framework proposal for training set of low-shot medical image data in cerebrovascular disease and comorbid depression

**DOI:** 10.1192/j.eurpsy.2025.1669

**Published:** 2025-08-26

**Authors:** A. Starcevic, B. Vucinic, F. Starcevic, B. Starcevic, M. Stojanovic

**Affiliations:** 1 Anatomy; 2 University of Belgrade; 3 University Clinical Center of Belgrade, Belgrade; 4 Private practice Ortodent, Paracin, Serbia

## Abstract

**Introduction:**

Cerebrovascular diseases, including white matter hyperintensities (WMLs), are the leading cause of functional disability and comorbid depression. Accurate and timely detection of WMLs is crucial for understanding their pathophysiology, progression, and treatment outcomes of both vascular and depressive symptomatology. Recent advancements in artificial intelligence (AI) and machine learning have facilitated the development of automatic WML detection methods, enhancing efficacy and accuracy in quantifying, localising, and evaluating lesion characteristics.

**Objectives:**

The aim of this pilot study was to propose the framework for training set of low-shot medical image data in cerebrovascular disease and comorbid depression as novel artificial intelligence (AI) neuroimaging techniques, combined with quantitative measurements can be used to investigate and understand the implications of WMLs on brain structure and function.

**Methods:**

We included 25 subjects, 15 with WMLs and 10 controls. Depressive symptoms were assessed by psychiatrist using Hamilton Depression Rating Scale (HDRS) and clinical interview. The neuroimaging data were collected from Magnetic Resonance Department University Clinical Center, Medical faculty, University of Belgrade, Serbia. All images were aligned with MNI space to normalize their intensity. Manual delineation and brain segmentation was performed with 3D slicer software using structural T1v and T2FLAIR images. Statistical analysis was performed to determine the correlation of lesion volume with the total brain volume (TBV) and to compare the TBV in patients with lesions and controls, in an effort to find possible morphometric correlates that may aid in automatic lesion detection and predicting the spacial pattern of WMLs.

Adding to the proposed framework, we have investigated statistical correlates in subjects with WMLs and controls. A statistical analysis was conducted to compare TBV in subjects with WMLs and controls and correlation between WML volume and TBV. The significance of the observed differences is determined, providing insights into the impact of WMLs on TBV.

**Results:**

The average WML volume was 197±154.18 mm^3^, with the smallest volume measured at 28.92 mm^3^ and the largest at 552.8 mm^3^. A significant difference in TBV was observed between patients with WMLs and controls (p-value=0.00216, p<0.05), and a weak negative correlation was found between WML volume and TBV (ρ=–0.167).

**Conclusions:**

Our findings demonstrate reduced TBV in patients with WMLs and depression and emphasize the importance of considering parameters such as TBV in evaluating WMLs. Assessing the impact of WMLs on TBV can provide valuable insights into the progression and severity of cognitive decline and functional impairment but also impact on training dataset preprocessing preparation for AI framework on low shot medical images in cerebrovascular disease.

**Disclosure of Interest:**

None Declared

